# Development and validation of a clinical model (DREAM-LDL) for post-stroke cognitive impairment at 6 months

**DOI:** 10.18632/aging.203507

**Published:** 2021-09-10

**Authors:** Yi Dong, Mengyuan Ding, Mei Cui, Min Fang, Li Gong, Zhuojun Xu, Yue Zhang, Xiuzhe Wang, Xiaofeng Xu, Xueyuan Liu, Gang Li, Yuwu Zhao, Qiang Dong

**Affiliations:** 1 Department of Neurology, Huashan Hospital, State Key Laboratory of Medical Neurobiology and MOE Frontiers Center for Brain Science, Fudan University, Shanghai, China; 2 Department of Neurology, The Tenth People’s Hospital Affiliated to Tongji University, Shanghai, China; 3 Department of Neurology, The East Hospital Affiliated to Tongji University, Shanghai, China; 4 Department of Neurology, The Six People’s Hospital Affiliated to Shanghai Jiao Tong University, Shanghai, China

**Keywords:** post-stroke cognitive impairment, acute ischemic stroke, the Montreal cognitive assessment, risk factors, low-density lipoprotein cholesterol

## Abstract

Introduction: This multicenter, retrospective study assessed the prevalence of post-stroke cognitive impairment (PSCI) 6 months after acute ischemic stroke (AIS) and its risk factors to build a bedside early predictive model for PSCI using the Montreal Cognitive Assessment (MoCA).

Methods: Records of consecutive patients with AIS treated at 4 stroke centers in Shanghai had MoCA assessments within 2 weeks after AIS onset and 6 months later were reviewed. Prevalence of PSCI (MoCA<22) was calculated and risk factors were identified by multivariate logistic regression analysis. The modeling and validation and identified risk factors were included in a predictive model using multivariate regression.

Results: There were 383 patients included and prevalence of PSCI 6 months after AIS was 34.2%, significantly lower than prevalence of patients with acute cognitive impairment (49.6%). Aging, less education, higher glucose level and severe stroke were PSCI risk factors, while level of low-density lipoprotein cholesterol (LDL-C) had a paradox effect on the risk of PSCI. 40.0% of the patients with cognitive impairment at acute phase reverted to normal, and patients with LDL-C 1.8-2.5 mmol/L were more likely to revert. The predictive model we built, DREAM-LDL (Diabetes [fasting blood glucose level], Rating [NIHSS], level of Education, Age, baseline MoCA and LDL-C level), had an AUROC of 0.93 for predicting PSCI at 6 months.

Conclusion: PSCI was common among AIS patients 6 months after AIS. We provided a practical tool to predict PSCI based on MoCA and risk factors present during acute phase of AIS.

## INTRODUCTION

Cognitive impairment is common in adults after stroke, which might increase the disability and mortality in post-stroke patient as well [1, 2]. Post-stroke cognitive impairment (PSCI) refers to cognitive impairment that develops after a stroke without any indications of major pre-stroke cognitive decline [3]. Prevalence of PSCI varies among different reports, ranging from 20% to 82% [3–6]. Because PSCI might recover due to rehabilitation and neuroplasticity during the first few months after stroke [3, 7], while less improvement would be found after 6 months [8, 9]. Therefore, it is essential to identify stroke survivors with high risk of developing PSCI as early as possible, so that early preventive and treatment measures could be taken to prevent or delay PSCI [1–3].

As a screening tool, the Montreal Cognitive Assessment (MoCA) is sensitive in detecting early PSCI. Though it was reported that MoCA score was a good predictor of PSCI in 6-9 month after stroke [4, 10], some risk factors such as age, level of education, medical history of hypertension, diabetes mellitus, smoking and atrial fibrillation reported, were associated with higher risk of PSCI [3, 11, 12]. However, the role of MoCA results in acute phase or any other bedside tool for PSCI risk is still lacking.

The aim of this study was to identify factors associated with 6-month PSCI. Using the characteristics and features in the acute stage of AIS, to build an easy and practical predictive model for PSCI in Chinese population would be helpful in identifying high-risk patients as early as the acute phase of AIS. In addition, there was of interest to explore the potential subgroup of patients with cognitive impairment at acute phase who returned to normal cognitive performance (“reverters”) 6 months after AIS onset.

## MATERIALS AND METHODS

### Study design and patients

This study was a multicenter retrospective study, which was approved by the Huashan Hospital Institutional Review Board (IRB protocol number V2.0; ethical approval number KY2017-201) and of each participating center before the study began. The trial was conducted in accordance with the guiding principles of the Declaration of Helsinki. All patients or their caregivers gave written informed consent before data collection.

The records of consecutive patients with confirmed AIS treated at 4 regional stroke centers from June 2017 to February 2018 were retrospectively reviewed. AIS was diagnosed by a neurologist according to the World Health Organization definition. Patients with AIS were provided with a MoCA assessment both within 2 weeks after AIS onset (baseline MoCA) and 6 months after AIS (6-month MoCA) [13]. Patients with pre-existing cognitive impairment before AIS according to their medical records and patients with severe cognitive impairment or aphasia who might be unable to undertake MoCA assessment were excluded. Then those patients’ medical records were further extracted including their demographic, baseline characteristics and clinical information such as the National Institutes of Health Stroke Scale (NIHSS) score [8, 10, 12], the subtype of stroke classification [8, 12] and the Oxfordshire Community Stroke Project (OCSP) classification were also collected [14].

### Outcome measures

A patient with a baseline MoCA score < 22 was considered to have acute cognitive impairment, and a patient with a 6-month MoCA score <22 was diagnosed as PSCI [10]. An additional point was added to a patient's MoCA total score when his/her education was less than 12 years [10]. The scores of the 7 MoCA subdomains (visuospatial/executive, naming, attention, language, abstraction, delayed recall and orientation) [13] were also recorded, separately. Additionally, the changes of patients’ 6-month MoCA scores were assessed for all patients [13].

The number and percentage of patients with acute cognitive impairment who recovered and had normal cognitive performance 6 month after AIS (MoCA ≥22) (“reverters”) were calculated and factors associated with the reverters were determined.

### Statistical analysis

Categorical data was described with number (n) and percentage (%) while normally distributed continuous data was described with mean ± standard deviation (SD) and non-normally distributed continuous data was described with median (interquartile range [IQR]). Prevalence of acute cognitive impairment and 6-month PSCI were calculated with the number of patients with cognitive impairment as numerator and the number of all included patients as denominator, and 95% confidential interval (CI) was estimated with Poisson distribution. Comparisons between two groups were performed using the chi-square test or Fisher’s exact test for categorical data, the Student t test for normally distributed continuous data and the Mann-Whitney U test or Wilcoxon rank sum tests (when appropriate) for non-normally distributed continuous data. Factors associated with PSCI were studied using univariate analysis entering patients’ baseline demographic and clinical characteristics. We put most variables with p<0.1 into the multivariate logistic regression and interactions would be tested before. The results were presented as odds ratios (ORs) with 95%CI. Statistical analyses were performed using the Stata V15.1(College Station, TX, USA). All tests were two-tailed, and a *P* value < 0.05 was considered statistically significant.

### Modeling and validation

The risk factors associated with PSCI at 6 months identified by the multivariate analysis were included in multivariate logistic regression analysis based on data from patients from 2 of the 4 centers (Huashan Hospital and the Tenth People’s Hospital) to build a predictive model for predicting a patient’s risk of developing PSCI 6 months after AIS. A scoring system (point 1-3) was used to score each risk factor in the model based on their OR and 95% CI. The model’s discriminatory ability was assessed with receiver operating characteristic (ROC) analysis using the area under the curve (AUC), and the Hosmer–Lemeshow test was used to confirm that the observed event rates match expected event rates in subgroups of the modeling population and to assess calibration of the model. Internal cross-validation of the regression model between parameters of the DREAM-LDL Scale score and 6-month cognitive impairment was based on 1,000 bootstrap replicates. Estimated mean accuracy and 95% confidence intervals would be analyzed. External validation of our predictive model was done in a separate cohort, another 2 of the 4 hospitals (East Hospital and Sixth People’s Hospital).

## RESULTS

### Prevalence of PSCI 6 months after AIS and its risk factors

**Figure 1:**
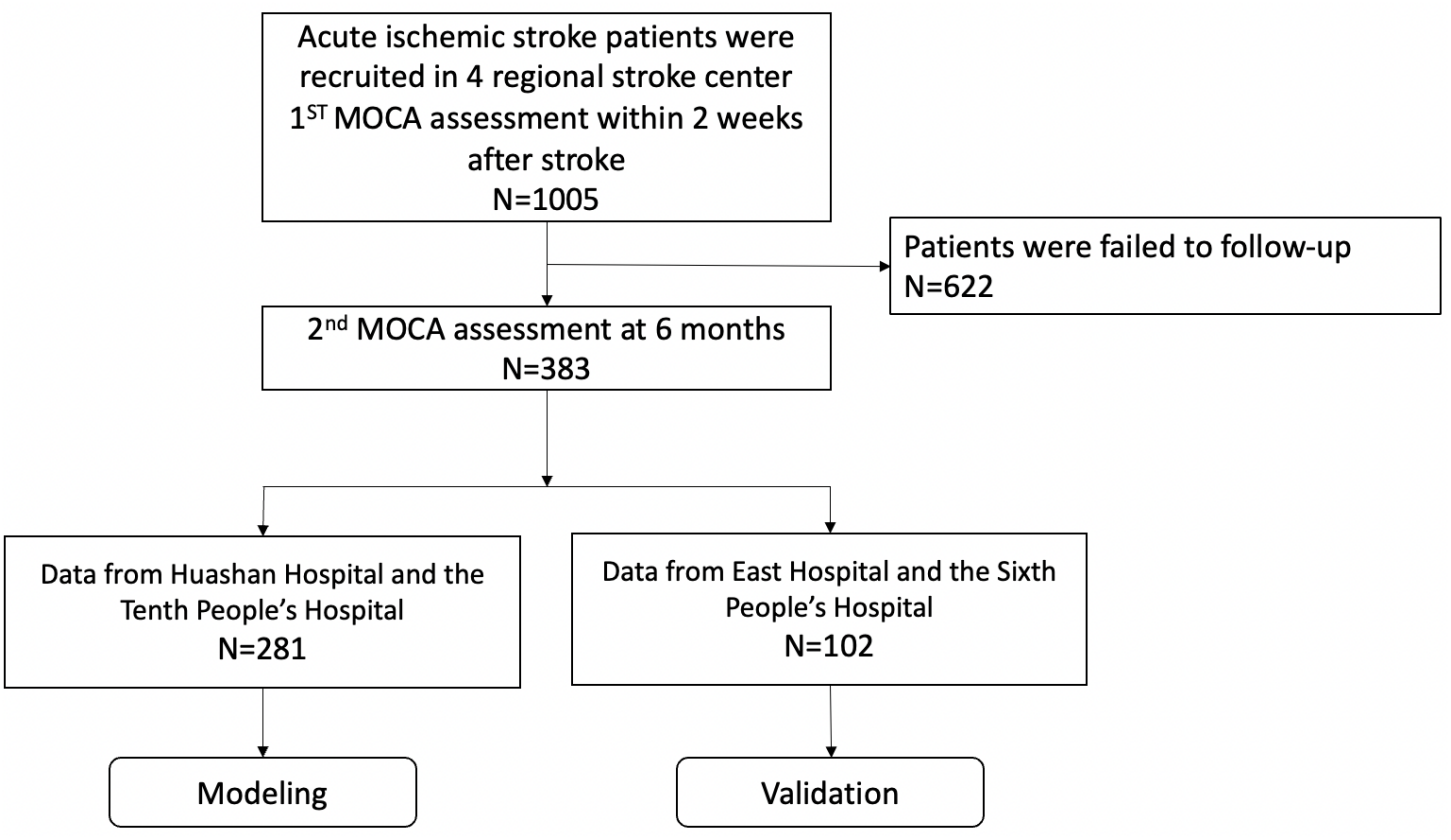
Study flowchart.

The study flow diagram was illustrated in Figure 1 total of 383 AIS patients were included in this study. They had a median age of 63 (interquartile range [IQR]: 56-70) years, and 75.46% of them were male. Most of them had mild or moderate stroke (NIHSS≤15, 88.77%).

Of the 383 patients, 131 patients had PSCI 6 months after their AIS onset and the prevalence of PSCI is 34.2% (95%CI 29.5%-39.1%). Demographics and baseline characteristics of the PSCI and non-PSCI patients at 6 months were described in Table 1. Univariate analysis revealed that compared to the non-PSCI population, the PSCI population had higher percentage of patients who were elder, female and more severe stroke and with fewer years of education. The PSCI population also had a significantly different distribution of patients with LDL- C level <1.8mmol/L, 1.8-2.5 mmol/L and ≥2.6mmol/L (Supplementary Figure 1).

**Table 1:** Patient demographic and baseline characteristics.

**Variables**	**All patients (N=383)**	**Non-PSCI (N=252)**	**PSCI (N=131)**	*P* value*
Age, years, median (IQR)	63.00 (56.00-70.00)	62.00 (56.00-69.50)	64.00 (56.00-71.00)	0.063
63<Age<80, n (%)	93 (24.28)	58 (21.64)	35 (30.43)	0.140
≥80, n (%)	102 (26.63)	64(23.88)	38 (33.04)	
Male, n (%)	289 (75.46)	211 (78.73)	78 (67.83)	0.023*
BMI, kg/m^2^, median (IQR)		24.22 (22.49-26.45)	23.48 (21.54-25.39)	0.058
Level of education				0.002*
≤5 years, n (%)	63 (16.45)	33 (12.31)	30 (26.09)	
>5 and ≤9 years, n (%)	140 (36.55)	95 (35.45)	45 (39.13)	
>9 and ≤12 years, n (%)	116 (30.29)	91 (33.96)	25 (21.74)	
>12 years, n (%)	64 (16.71)	49 (18.28)	15 (13.04)	
Severity of stroke, median [interquartile]	11[2-14]	11[2-14]	11[3-14]	0.016*
Mild-Moderate (NIHSS≤8), n (%)	199 (51.96)	137 (51.11)	62 (65.35)	0.985
Moderate (8<NIHSS<15), n (%)	141(36.81)	87(32.46)	54 (28.71)	
Severe (NIHSS≥15), n (%)	43 (11.23)	28 (6.28)	15 (5.94)	
TOAST classification				0.120
Large artery atherosclerosis, n (%)	241 (63.09)	174 (64.93)	67(58.77)	
Cardioembolism, n (%)	22 (5.76)	15 (5.60)	7(6.14)	
Small vessel occlusion, n (%)	88 (23.04)	61 (22.76)	27 (23.68)	
Other cause, n (%)	11 (2.88)	9 (3.36)	2 (1.75)	
Unknown cause, n (%)	20 (5.24)	9 (3.36)	11 (9.65)	
OCSP classification				0.112
TACI, n (%)	12 (3.14)	5 (1.87)	7 (6.14)	
PACI, n (%)	218 (56.62)	152 (56.72)	66 (57.89)	
POCI, n (%)	21 (5.50)	17 (6.34)	4 (3.51)	
LACI, n (%)	131 (34.39)	94 (35.07)	37 (32.46)	
FBG, mmol/L, median (IQR)	5.40(4.80-7.16)	5.30(4.80-6.50)	5.60(4.90-7.53)	0.156
5.4<FBG<7.1, n(%)	101(37.62)	63(23.51)	38(33.04)	0.080
≥7.1, n(%)	105(39.04)	64(23.88)	41(35.65)	
LDL-C, mmol/L, median (IQR)	2.64 (2.11-3.19)	2.65 (2.14-3.22)	2.58 (1.82-3.23)	0.201
<1.8, n (%)	63 (16.62)	35 (13.21)	28 (24.56)	0.014*
1.8≤LDL-C<2.6, n (%)	122 (32.19)	93 (35.09)	29 (25.44)	
≥2.6, n (%)	194 (51.19)	137 (51.70)	57 (50.00)	
Medical history				
Hypertension, n (%)	185 (48.30)	134 (50.00)	51 (44.35)	0.310
Diabetes, n (%)	102 (26.63)	66 (24.63)	36 (31.30)	0.175
Dyslipidemia, n (%)	7 (1.83)	5(1.87)	2(1.74)	0.320
Atrial fibrillation, n (%)	16 (0.41)	8(0.32)	8(0.61)	0.175
Prior stroke, n (%)	8 (2.09)	8(2.99)	0(0.00)	0.055
Myocardial infarction, n (%)	2 (0.052)	2(0.75)	0(0.00)	0.489

*P value referred to comparison between PSCI and non-PSCI patients.

PSCI, post-stroke cognitive impairment; IQR, interquartile range; FBG, fasting blood glucose; LDL-C, low density lipoprotein (LDL) cholesterol; NIHSS, The National Institutes of Health Stroke Scale; TOAST, Trial of ORG 10172 in Acute Stroke Treatment; OCSP, The Oxfordshire Community Stroke Project; TACI, total anterior circulation infarct; PACI, partial anterior circulation infarct; POCI, posterior circulation infarct; LACI, lacunar circulation infarct.

Multivariate logistic regression analysis were listed in Supplementary Table 1 and only MoCA performance was independently related to the risk of PSCI at 6 months (Table 2).

**Table 2:** Multivariate logistic regression analysis of risk/protective factors for patients developing PSCI 6 month after acute ischemic stroke.

**Variables**	**OR**	**95% CI**		**P value**	
**Baseline MoCA**	0.74	0.69~0.78			<0.001
**Level of education** (<6yr as reference, as categorical variables)					0.219
6-9 years	1.15	0.50~2.63			
9-12 years	0.72	0.30~1.71			
≥12 years	0.70	0.27~1.89			
**Age**(as categorical variables)					0.572
≤63 years old	As refer			
63<Age<80 years old	1.30	0.73~2.32			
≥80 years old	0.94	0.29~3.03			
**FBG** (as categorical variables)				
≤5.4mmol/L	As refer				0.482
5.4mmol/L<FBG<7.1mmol/L	1.49	0.76~2.95			
≥7.1mmol/L	1.27	0.64~2.53			
**LDLC level** (LDL-C<1.8mmol/L or LDL-C≥2.6mmol/L as reference)					0.327
0.3342.6>LDL-C≥1.8mmol/L	0.76	0.43~1.33			
**Severity of Stroke (NIHSS, points**, as categorical variables)					0.876
Moderate (8<NIHSS<15), n (%)	1.11	0.57~2.14			
Severe (NIHSS≥15), n (%)	0.90	0.43~1.89			

PSCI, post-stroke cognitive impairment; OR, odd ratio; CI, confidential interval; LDL-C, low density lipoprotein (LDL) cholesterol.

Spearman’s rank correlation indicated that a patient’s baseline MoCA score was negatively correlated to the risk of PSCI at 6-month (rs=-0.61, P<0.001).

### Temporal changes of the patients’ cognitive performance

According to these patients’ baseline MoCA assessments taken within 2 weeks of their AIS onset, 190 of the 383 patients had acute cognitive impairment (49.6%), the prevalence of acute phase was significantly higher than the prevalence of PSCI 6 months after AIS (34.2%). Of the 190 patients with acute/subacute cognitive impairment, 76 (40.0%) patients recovered (reverter) and had normal cognitive status 6 month after AIS, while 17 (8.9%) with normal cognitive status at baseline deteriorated and had PSCI 6 month after AIS.

The patients’ general and domain-specific changes in their cognitive performance were described in Table 3. Among them, the mean MoCA score of patients with PSCI at 6 months (N=131) had insignificant improvement from baseline (15.78±5.60 at baseline vs 16.13±4.40 at 6 months, *P*=0.0519), while there was significant improvement in the 252 non-PSCI patients’ mean MoCA score (24.03±4.17 at baseline vs 26.58±2.27 at 6 months, *P*<0.001). For patients with PSCI, their visuospatial/executive and delayed recall improved significantly, naming and language abilities deteriorated while their attention, abstraction and orientation remained stable. Overall, non-PSCI patients performed significantly better than PSCI patients in all of the 7 subdomains of MoCA and also MoCA total scores at baseline as well as 6 months after their AIS (All P<0.001).

**Table 3:** Temporal changes of MoCA total and subdomain scores in patients with acute ischemic stroke.

**Domains**	**Scoring range**	**All patients (N=383)**				**PSCI (N=131)**				**Non-PSCI (N=252)**			**t-test** **values^#^**	P value*
		**Baseline**	**6-month**	**t-test values**		**Baseline**	**6-month**	**t-test** **values**		**Baseline**	**6-month**	**t-test** **values**		
Visuospatial/ executive	0-5	2.91 ± 1.64	3.39 ± 1.48	6.21		1.81 ± 1.43	2.16 ± 1.47	2.31		3.48 ± 1.44	4.02± 1.01	6.30	14.54	<0.001
Naming	0-3	2.54 ± 0.80	2.44 ± 0.80	-2.37		2.10 ± 1.00	1.90 ± 1.01	-2.20		2.76 ± 0.55	2.72 ± 0.49	-1.07	10.91	<0.001
Attention	0-6	4.91 ± 1.37	5.02 ± 1.27	1.66		4.02 ± 1.62	4.07 ± 1.51	0.37		5.37 ± 0.96	5.52 ± 0.74	2.01	12.46	<0.001
Language	0-3	1.97 ± 1.07	1.96 ± 1.01	-0.24		1.26 ± 1.02	1.07 ± 0.92	-1.85		2.35 ± 0.88	2.42 ± 0.69	1.19	16.04	<0.001
Abstraction	0-2	1.15 ± 0.79	1.46 ± 0.91	6.09		0.72 ± 0.73	0.79 ± 0.75	1.00		1.37 ± 0.72	1.81 ± 0.79	1.00	12.06	<0.001
Delayed recall	0-5	1.95 ± 1.71	2.91 ± 1.74	10.52		0.92 ± 1.44	1.31 ± 1.48	2.58		2.49± 1.60	3.75 ± 1.19	11.36	17.55	<0.001
Orientation	0-6	5.29 ± 1.29	5.43 ± 1.17	2.07		4.70 ± 1.63	4.67 ± 1.67	-0.21		5.6 ± 0.93	5.82 ± 0.43	3.57	10.30	<0.001
Total	0-30	21.55 ± 5.99	23.44 ± 5.69	9.20		16.41 ± 5.72	16.85 ± 4.54	1.11		24.23 ± 4.07	26.87± 2.01	11.84	29.83	<0.001

The t tests were performed to compare the baseline performance to the 6-month performance in all patients, PCSI population and non-PSCI population, respectively.

^#^t tests were also performed between PCSI population and non-PSC population at 6months.

*P value referred to comparison between PSCI and non-PSCI patients at 6 months.

Variables were expressed as mean ± standard deviation (SD).

MoCA, the Montreal Cognitive Assessment.

In addition, 76 the 190 patients (40.0%) with cognitive impairment at acute phase recovered and reverted back to have normal cognitive status at 6-month after AIS. The reverters’ mean MoCA score improve to 25.71±1.89 at 6-month from the baseline 19.34±3.23 (P<0.001). Multivariate logistic regression in patients with acute cognitive impairment revealed that patient with appropriate LDL level was more likely to be reverters (OR 1.79, 95%CI: 1.00~3.22, P=0.041).

### Predictive model building and validation

**Figure 2:**
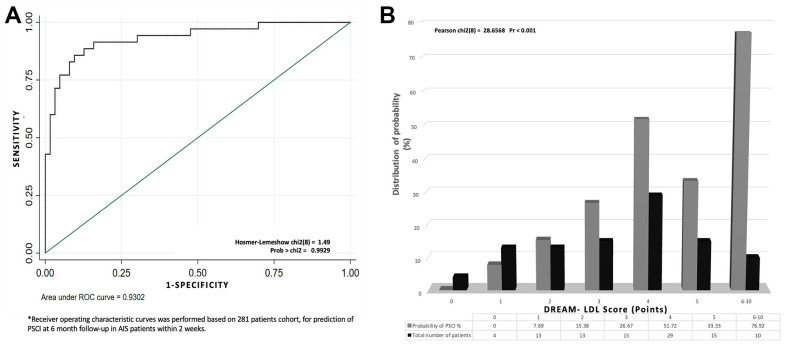
**The establishment and validation of the DREAM-LDL scale.** (**A**) Receiver operating characteristic (ROC) curve based on a cohort of 281 patients for identifying AIS patients at risk of mid-term PSCI as early as within 2 weeks of AIS onset. (**B**) The DREAM-LDL scale was validated in another cohort of 102 AIS patients.

The internal validation model was based on 281 patients from Huashan Hospital and the Tenth People’s Hospital. The predictive model, DREAM-LDL, included 6 variables: diabetes (fasting blood glucose level), rating (severity of stroke as reflected by NIHSS at admission), level of education, age, baseline MoCA and LDL-C level. DREAM-LDL had a risk score ranging from 0 to 10 (Table 4). We scored these risk factors according to their odds ratio (Supplementary Tables 1, 2). The DREAM-LDL had an AUROC of 0.93, its Hosmer-Lemeshow chi^2^(8) was 1.49 (Figure 2A). Accuracy of the derivation model based on 1,000 bootstrap replicates was 75.7% (66.4%– 84.9%). A 3-tier scoring system was used to evaluate a patient’s risk of having PSCI 6 months after AIS. A patient with a score of 0-2 points was considered to have low risk, a patient with a score of 3-5 points was considered to have a moderate risk and a patient with 6 points or high was considered to have a high risk.

**Table 4:** DREAM-LDL: a clinical model for the risk of PSCI at 6-month.

**Risk factors**	**Scores**
**Diabetes(FBG, mmol/L)**	
FBG>7.1	2
7.1>FBG>5.4	1
**Rating(Severity of Stroke, NIHSS)**	
NIHSS8≥15	2
15≥NIHSS≥8	1
**Level of Education**	
No more than 9 years	2
No more than 12 years	1
**Age**	
> 80 years old	2
> 63 years old	1
**Baseline MoCA<22**	2
**Inappropriate LDL-C (1.8-2.6mmol/L)**	1

DREAM LDL scale predict PSCI after stroke is ranged from 0-10 points. Low risk is as 0-2 points. Moderate risk is as 3-5 points. High risk is defined as 6 or higher.

FBG, Fasting blood glucose; NIHSS, The National Institutes of Health Stroke Scale; LDL-C, low density lipoprotein (LDL) cholesterol; MoCA, Montreal Cognitive Assessment.

External validation of the model was performed on the 102 patients from the 2 of the 4 hospitals in the study (East Hospital and the Sixth People’s Hospital). 7.89% of the patients with a score of 0-2 points, 40.68% of the patients with a score of 3-5 points and 78.82% of the patients with a score of 6 points or higher had PSCI 6 months after AIS (Figure 2B). The higher the DREAM-LDL score was, the higher the likelihood of PSCI at 6-month. There was no statistical difference in AUC-ROC between the derivation and validation cohorts. Additionally, we compared the performance of DREAM-LDL in reverters and non-reverters in Supplementary Figure 2, which showed no statistical difference.

## DISCUSSION

Our finding of PSCI prevalence of 34.2% was consistent with the mid-term PSCI prevalence reported by these previous studies [1, 4, 6, 15]. Based on this AIS database, we found PSCI was common at 6-months and that old age, fewer year of education, history of atrial fibrillation and acute cognitive impairment were risk factors for PSCI. However, the LDL-C level of 1.8-2.5mmol/L was a likely protective factor against PSCI. We established and validated a bedside predictive tool, DREAM-LDL, which could be helpful in early detection of PSCI and potential reverters.

Our study showed that LDL-C played a paradox role in whether a patient was at risk of PSCI 6 months after AIS (Supplementary Figure 1). Previous studies produced conflicting results on relationship between LDL-C level and cognitive ability [16–32]. According to the National Lipid Association recommendations for patient-centered management of dyslipidemia, the desirable LDL-C level was <1.8mmol/L (100mg/dL) for people at very high risk of atherosclerotic cardiovascular disease (ASCVD) and <2.6mmol/L (130mg/dL) for those without clinical evidence of ASCVD or other very high-risk conditions [33]. Our result indicated that LDL-C in the upper range of normal is most suitable for reducing the risk of PSCI as well as increasing the likelihood of patients with acute cognitive impairment to recover.

Therefore, our DREAM-LDL bedside tool might be helpful to identify AIS patients at risk of mid- and long-term PSCI as early as possible. Most variables included in our DREAM-LDL risk score were identified by previous studies as important risk factors for PSCI [3, 11–13, 34–39]. Several current available predictive tools input some neuroimaging and techniques [3, 12, 38, 39]. The SIGNAL_2_ risk score consists of age stages, education<6 years, global cortical atrophy stages, Fazekas stages, non-lacunar cortical infarct stages, chronic lacunes≥2 and intracranial stenosis [12]. The CHANGE risk score includes age, education, cortical atrophy, acute cortical non-lacunar infarcts, white matter hyperintensities (WMH) and chronic lacunes [3]. The GRECogVASC cognitive risk score consists of NIHSS≥7 at admission (≥7), multiple strokes, adjusted MMSE≤27, and Fazekas score≥2 [37]. Finally, Ding et al. developed a PSCI risk model consisting of age, years of education, periventricular hyperintensity grading, diabetes mellitus and the number of acute non-lacunar infarcts as variables [36]. Neuroimaging information is needed for all of these 4 models [3, 12, 36, 37]. Our risk score does not rely on any neuroimaging information, therefore, is simple and easy to administer in intensive stroke unit. Of course, the model needs to be tested and further validated in other AIS patient populations.

Our study has certain limitations. First, it was a 6-month study, therefore how these patients’ cognitive performance would evolve beyond 6 months could not be evaluated. Secondly, only very few of the included patients (11.23%) suffered severe stroke and as such, the effect of severity of stroke on the risk of PSCI and patients’ likelihood to recover could not be adequately evaluated. However, it was the nature or limit of a retrospective study. Thirdly, we used a MoCA cutoff value of 22 in the current study for estimating prevalence of PSCI and for building the predicting model for PSCI. which was lower than mean MoCA score of old healthy subjects reported by many studies [40–45]. Fourthly, the MoCA testing was originally designed for detecting mild cognitive impairment, but not for PSCI and therefore it does not include assessment of some of the cognitive deficits commonly seen after stroke such as aphasia, neglect, visual loss, apraxia and reading/writing problems [46–48]. However, up till now, the MoCA test is still the commonly used bedside screening tool for PSCI. Therefore, it should be assumed that AIS patients with severe stroke symptoms incapable of completing the MoCA testing were at high risk of developing PSCI.

In conclusion, PSCI was common among AIS survivor 6 months after AIS. Our finding provided a tool for early identification of AIS patients at high risk of developing PSCI.

## Supplementary Material

Supplementary Figures

Supplementary Tables
