# Supplementary material for: Development and validation of a clinical model (DREAM-LDL) for post-stroke cognitive impairment at 6 months
Source: Aging (Albany NY). 2021 Sep 10;13(17):21628–41. doi: 10.18632/aging.203507 (PMC8457606; doi:10.18632/aging.203507)
Supplement: Supplementary Figures [file aging-13-203507-s001.pdf]

SUPPLEMENTARY FIGURES

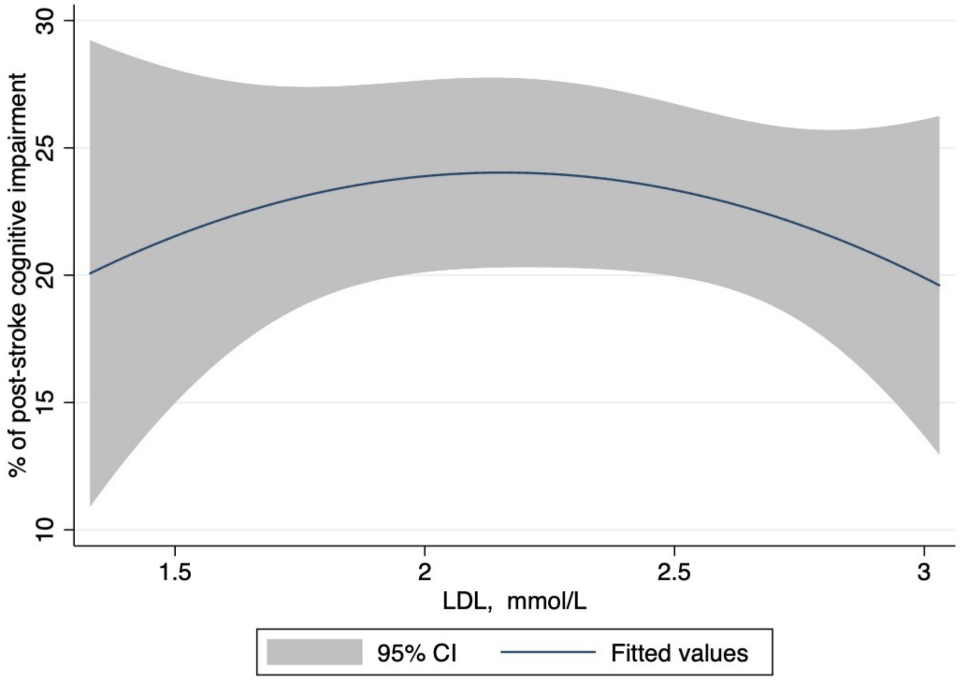

Supplementary Figure 1. The correlation between LDL level at baseline and risk of PSCI at 6 months after stroke.

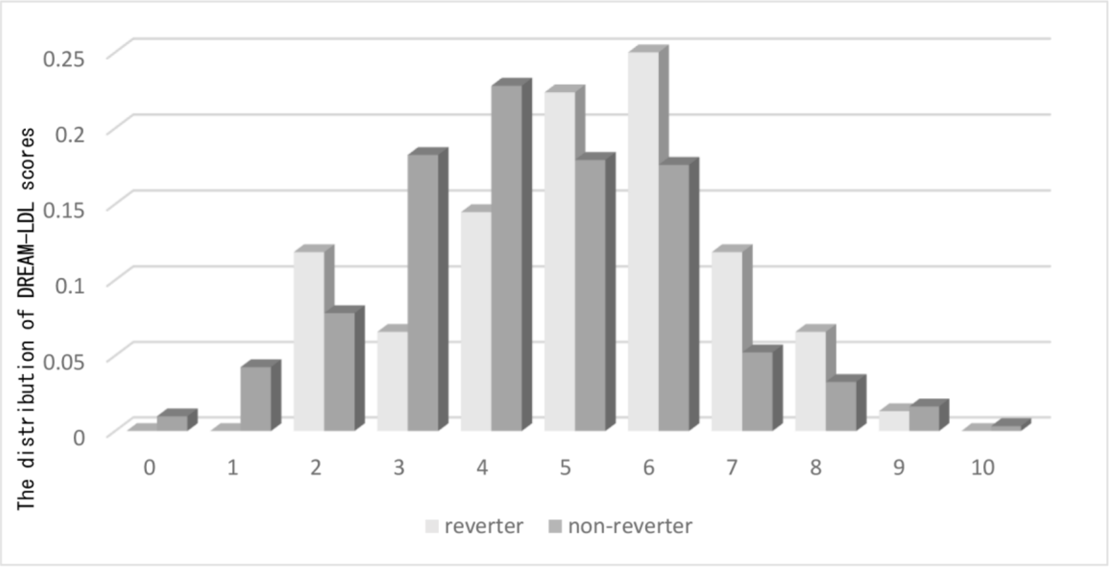

Supplementary Figure 2. Comparison of the distribution of DREAM-LDL scores between reverter and non-reverter.
