# Supplementary material for: Development and validation of a clinical model (DREAM-LDL) for post-stroke cognitive impairment at 6 months
Source: Aging (Albany NY). 2021 Sep 10;13(17):21628–41. doi: 10.18632/aging.203507 (PMC8457606; doi:10.18632/aging.203507)
Supplement: Supplementary Tables [file aging-13-203507-s002.pdf]

SUPPLEMENTARY TABLES

Supplementary Table 1. The risk factors of PSCI at 6-month in logistic regression model.

Variables	Univariable analysis			Multivariable analysis, as categorical variable		
	Odds ratio	95% CI		Odds ratio	95% CI	
**Age, each year**	1.08	1.00	1.04	1.15	0.66	2.02
>63yrs	1.38	0.91	2.12			
>80yrs	1.61	0.65	4.01			
**Level of education, each 3 years**	0.69	0.54	0.86			
More than 9 years	0.52	0.42	0.64	0.85	0.63	1.14
**MoCA, each point**	0.63	0.56	0.70	0.74	0.69	0.78
**Diabetes mellitus**	1.28	0.83	1.97			
Glucose level, 1 mmol	1.05	0.98	1.14	1.06	0.58	1.95
Glucose level>5.4mmol/l	1.49	0.97	2.29			
**Severity of Stroke**				1.00	0.67	1.38
Baseline NIHSS score,1 point	1.04	1.00	1.08			
NIHSS>8 points	1.65	1.04	2.63			
NIHSS>15 points	1.29	0.98	1.70			
**LDL level, 1 mmol/L**	1.02	0.96	1.09	0.76	0.43	1.33
Appropriate LDL(1.8-2.6mmol/L)	0.69	0.45	1.05			

Supplementary Table 2. Multivariate logistic regression analysis of the risk factors for PSCI at 6 months included in the predictive model for the patients from 2 of the 4 study centers (Huashan Hospital and the Tenth People's Hospital) (N=281).

Variables	Odds ratio	95% Confidential interval		P value
**Baseline MoCA**	15.454	8.466	-28.210	0.000
**Level of education**				
<9 years, n (%)	1.788	0.986	-3.240	0.056
9-11 years	1.825	0.908	-3.667	0.091
≥12 years	As refer			
**Age**				
≤63 years old	As refer			
64-79 years old	1.052	0.551	-2.010	0.878
≥80 years old	0.902	0.488	-1.669	0.743
**FBG**				
≤5.4mmol/L	As refer			
5.5-7.0 mmol/L	1.572	0.834	-2.963	0.162
≥7.1mmol/L	1.191	0.641	-2.213	0.581
**LDL-C**				
1.8-2.5 mmol/L	As refer			
<1.8mmol/L or ≥2.6mmol/L	1.364	0.774	--2.404	0.284
**Severity of Stroke (NIHSS, points)**				
Moderate (NIHSS 9-14), n (%)	1.060	0.610	-1.842	0.836
Severe (NIHSS≥15), n (%)	0.848	0.371	--1.939	0.697
Atrial fibrillation	1.172	0.638	- 2.154	0.638

FBG, fasting blood glucose level; OR, odd ratio; CI, confidential interval; LDL-C, low density lipoprotein (LDL) cholesterol; NIHSS, National Institution of Health Stroke Scale.
